# Poor survival of females with bladder cancer is limited to those aged 70 years or over: a population-wide linkage study, New South Wales, Australia

**DOI:** 10.1002/cam4.452

**Published:** 2015-04-27

**Authors:** Manish I Patel, Albert Bang, David Gillett, Rajkumar Cheluvappa, David P Smith

**Affiliations:** 1 Westmead Hospital, Discipline of Surgery, University of SydneySydney, NSW, Australia; 2 Cancer Research Division, Cancer Council NSWSydney, NSW, Australia; 3 Department of Surgery, Macquarie UniversitySydney, NSW, Australia; 4 Griffith Health Institute, Griffith UniversityNathan, Queensland, Australia

**Keywords:** Age, bladder cancer, gender, sex, survival

## Abstract

Although men are diagnosed with bladder cancer (BC) with a rate three times higher than women, women experience poorer survival. The cause of this gender difference is not clear. The aim of this study was to investigate the discrepancy in survival from BC by gender and explore potential explanations for the difference using a population-wide linkage study. Using the New South Wales (NSW) Central Cancer Registry, all invasive BC cases diagnosed between 2001 and 2009 were identified. Records were linked to the NSW Admitted Patient Data Collection (APDC), to retrieve treatment details, and to the Registry of Births Deaths and Marriages and Australian Bureau of Statistics to obtain death details. A total of 5377 new cases of BC were identified. No differences were identified in the proportions of patients presenting at different stages between genders. However, disease-specific survival (DSS) was worse for females compared to males with localized and regional disease (*P* < 0.05). This difference was only apparent in individuals aged ≥70 years and no difference was identified in those younger. Multivariable Cox-regression analysis of the cohort of individuals aged ≥70 years revealed that stage, age, comorbidity, and sex remained independent variables (*P* < 0.05) predicting DSS. In a population wide analysis, females aged 70 years or more suffer worse DSS compared to males. The differences are not accounted for by stage at presentation or comorbidity and are independent of age. BC in postmenopausal females may be biologically more aggressive.

## Introduction

Between 1970 and 2008, the incidence of bladder cancer (BC) in males and females residing in New South Wales (NSW) Australia has decreased primarily due to decreased smoking, although this trend may be partially artifactual due to coding practice changes 1. Over this time, male BC mortality rates have also decreased, however female mortality rates have not 1–3. The reason for such discrepancy between genders is not understood.

Demographic heterogeneity is well recognized in patients with BC, however the effect of gender on BC incidence, stage at presentation, mortality, and survival time has not been well described. Survival from BC has been reported to be worse for females compared to males 4–10, despite the fact that BC is 3–4 times more common in males than females. However, this finding is controversial. Some reports have shown no difference in survival between genders following radical cystectomy 11, while other reports indicate that females lose substantially more years of life from BC mortality compared to men 12.

Suggested reasons for worse survival include late presentation 6, disparities in treatment 13 or intrinsic biological differences 14. The aim of this study was to investigate the discrepancy in survival from BC by gender and explore potential explanations for the difference using a population-wide linkage study.

## Methods

Data for NSW residents with a diagnosis of BC were obtained from the NSW Central Cancer Registry (CCR). Operational details of the Registry have been described previously 3. In brief, notifications to the CCR of invasive BC (ICD-10 code: C67) diagnosed in NSW are mandated from pathology laboratories, hospitals and other treatment centers under the NSW Public Health Act 1991 3. Noninvasive and in situ BCs are not registered and not included in this study. For this study, all registered cases diagnosed between January 2001 and December 2009 were included. Death information was obtained by electronic linkage of the records from the CCR with NSW death records from the Registry of Births Deaths and Marriages to ascertain vital status (January 2001–December 2009) and Australia Bureau of Statistics to ascertain cause of death (January 2001–December 2007). All (100%) of the patients were able to be linked. Hospital admission and treatment details were retrieved by linkage to the NSW Admitted Patient Data Collection (APDC) for all hospital separations in NSW in the period January 2000–June 2009. Linkage was successful in 98% of cases. Hospital medical coders abstract individual patient information from medical records following the patient’s discharge from the hospital. This includes dates of admission and separation, procedures carried out and diagnoses relating to the hospital episode. Linkage of records in these data sets was carried out by the Centre for Health Record Linkage (CHeReL), using probabilistic matching carried out with ChoiceMaker software (ChoiceMaker Technologies Inc., New York, NY). This linkage was performed using name, address, date of birth, date of diagnosis, and hospital-recorded clinical information that identified cases common to all data sets. Clerical reviews for questionable matches were undertaken by trained staff within the CHeReL.

For the 74% of BC cases where data on degree of spread at diagnosis was available, cancers were categorized as localized, regional or distant. BC notified to the CCR with a 2001–2009 diagnosis and a recorded degree of spread was investigated in the present study. Eighty six (<2%) cases identified from autopsy or death records (“death certificate only”) were excluded because it was not possible to ensure that the date of diagnosis, recorded by the CCR as month and year of diagnosis, did not precede the date of death. All cases were classified by: (1) age at diagnosis categorized in decades with open-ended categories under 40 years and 80 years and over; (2) Charlson comorbidity index (CCI) (3) socioeconomic status (SES), using five ordinal categories derived from the residential Local Government Area-based Socioeconomic Index of Relative Disadvantage for Areas (SEIFA) 15; (4) Accessibility and remoteness of residence using five ordinal categories based on the Local Government Area classification of Accessibility/Remoteness Index of Australia (ARIA) 16; and (5) country of birth, categorized as Australian and New Zealand born, European, Asian and Other. Sixty-four cases (1.2%) with unknown country of birth were grouped with the Australian born for the purposes of the present study. Period of diagnosis was grouped into five periods for statistical analysis. (6) Degree of spread was classified as localized, regional distant and unknown and was based on the CCR classification of stage at first presentation. This was determined as the maximum extent of the cancer based on all reports and notifications dated within 4 months of diagnosis. This classification follows the international coding guidelines for summary stage adopted by the World Health Organisation and the International Association of Cancer Registries 17. Extent was grouped as localized (cancer was limited to bladder – Stage I and II), regional (cancer extended outside the bladder locally– stage III and some IV), distant (cancer in regional lymph nodes or distant metastases-stage IV) or an unknown degree of spread. Tumor grade information is not collected by the CCR and hence unavailable for analysis.

The percentages of cases (±standard error, SE) dying from BC within 5 years of diagnosis were calculated using Kaplan–Meier product-limit estimates 8,9. Differences were tested by the log rank test. Cases who were alive at the end of the study were censored on mid-December 2009. The percentage of patients diagnosed with and dying from BC by 5 years from diagnosis were presented by degree of spread for each sociodemographic characteristic, and for each diagnostic period.

No disease-specific survival (DSS) and overall survival (OS) differences were observed between the sexes in those aged less than 70 years so we performed bivariate and multivariate analysis using a Cox proportional hazards model of those patients aged ≥70 years at time of diagnosis. All covariates included have been reported in Table2 and the final model satisfied the proportional hazards assumption.

All analyses were carried out in SAS version 9.3 (SAS Institute Inc., Cary, NC) except the annual average percentage change figure calculated using Joinpont regression. NSW Population and Health Services Research Ethics Committee approved this study.

## Results

There were 5377 newly diagnosed cases of BC between 2001 and 2009 included in this study. Details of their characteristics are given in Table1. A total 72% of all cases were diagnosed in males and 28% in females. Death from BC in our patient cohort was substantially higher in women (40%) compared to men (30%) (*P* < 0.05) (Table1).

**Table 1:** Patient and disease characteristics of NSW residents diagnosed with bladder cancer in 2001–2009

	Males (%)	Females (%)	Total (%)
*N*	3891 (72.3)	1486 (27.7)	5377
Age			
Median	74	77	75
<50	121 (3.1)	47 (3.2)	168 (3.1)
50–59	390 (10.0)	97 (6.5)	487 (9.1)
60–69	847 (21.8)	229 (15.4)	1076 (20.0)
70–79	1401 (36.0)	501 (33.7)	1902 (35.4)
80+	1132 (29.1)	612 (41.2)	1744 (32.4)
Charlson comorbidity			
0	2270 (58.3)	890 (59.9)	3160 (58.8)
1	643 (16.5)	223 (15.0)	866 (16.1)
≥2	978 (25.1)	373 (25.1)	1351 (25.1)
Extent of disease			
Localized	1937 (49.8)	719 (48.4)	2656 (49.4)
Regional	675 (17.4)	256 (17.2)	931 (17.3)
Distant	253 (6.5)	145 (9.8)	396 (7.4)
Unknown	1026 (26.4)	366 (24.6)	1392 (25.9)
Histology			
Transitional cell	3555 (91.4)	1281 (86.2)	4836 (89.9)
Squamous cell	31 (0.8)	32 (2.2)	63 (1.2)
Adenocarcinoma	41 (1.1)	21 (1.4)	62 (1.2)
Others/unknown	264 (6.8)	152 (10.2)	416 (7.7)
Death			
Bladder cancer	1169 (30.0)	592 (39.8)	1761 (32.8)
Other causes	789 (20.3)	244 (16.4)	1033 (19.2)
Unknown	229 (5.9)	73 (4.9)	302 (5.6)
Total	2187 (56.2)	909 (61.2)	3096 (57.6)
Cystectomy			
Yes	610 (15.7)	195 (13.1)	805 (15.0)
Intravesical therapy			
Yes	502 (12.9)	123 (8.3)	625 (11.6)
Intravenous therapy			
Yes	311 (8.0)	88 (5.9)	399 (7.4)
Hospital of primary treatment			
Public	1524 (39.2)	644 (43.3)	2168 (40.3)
Private	2367 (60.8)	842 (56.7)	3209 (59.7)
Remoteness of residence (ARIA)			
Major cities	2643 (67.9)	1031 (69.4)	3674 (68.3)
Inner regional	966 (24.8)	348 (23.4)	1314 (24.4)
Outer regional/remote	282 (7.3)	107 (7.2)	389 (7.2)
Socioeconomic status SEIFA			
Least disadvantaged (Q1)	708 (18.2)	292 (19.7)	1000 (18.6)
Q2	629 (162)	233 (15.7)	862 (16.0)
Q3	872 (22.4)	321 (21.6)	1193 (22.2)
Q4	960 (24.7)	360 (24.2)	1320 (24.6)
Most disadvantaged (Q5)	722 (18.6)	280 (18.8)	1002 (18.6)
Country of birth			
Australia/NZ	2444 (62.8)	1084 (73.0)	3528 (65.6)
Europe	1086 (27.9)	289 (19.5)	1375 (25.6)
Asia	92 (2.4)	27 (1.8)	119 (2.2)
Other	269 (6.9)	86 (5.8)	355 (6.6)
Treatment details			
Matched	3816 (98.1)	1468 (98.8)	5284 (98.3)
Missing	75 (1.9)	18 (1.2)	93 (1.7)

**Figure 1:**
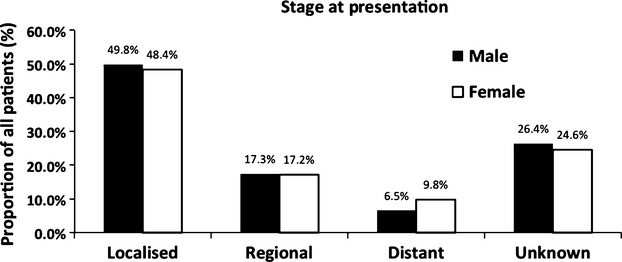
Proportion of patients presenting with localized, regional, distant, and unknown stage of bladder cancer.

**Figure 2:**
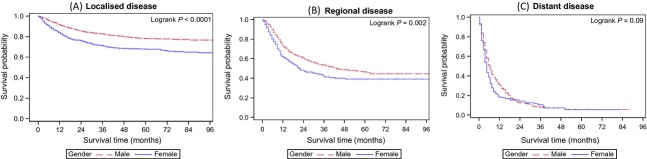
Kaplan–Meier disease-specific survival curves for localized (A), regional (B) and distant (C) disease by sex.3

To determine if late presentation in females and hence differences in stage at presentation was the cause of higher mortality, we analyzed stage at presentation by gender (Fig.1). Five-year DSS for localized, regional, and distant disease are 75.2% (95% CI: 73.3–77.0), 44.5% (95% CI: 40.8–48.2), and 5.4% (95% CI: 2.8–8.8), respectively. Individuals with unknown stage (26%), had the same male: female ratio, age distribution and DSS as those with known stage. There were no differences in the proportion of patients presenting with localized, regional, and distant disease between males and females (*P* > 0.05). Analysis of DSS between males and females indicated significantly worse survival in females with localized disease (Fig.2A) (*P* < 0.001) and regional disease (Fig.2B) (*P* = 0.002), but not with metastatic disease (Fig.2C) (*P* = 0.09). This difference was not explained by differences in histology; although women presented with higher incidence of squamous cell carcinoma (*P* < 0.001) this was minor (increase of 1.4%) and could not explain the survival difference (Table1). In addition, only minor, nonsignificant differences in treatment were identified between genders (use of cystectomy, intravesical treatments, and intravenous chemotherapy) which also did not explain the differences in survival.

**Figure 3:**
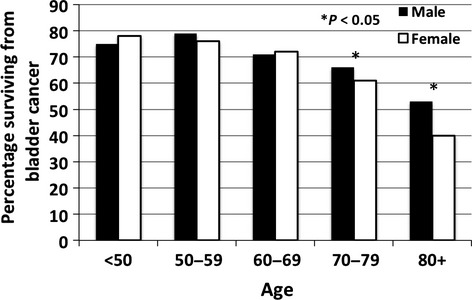
Five-year disease-specific survival for males and females by age group.

**Figure 4:**
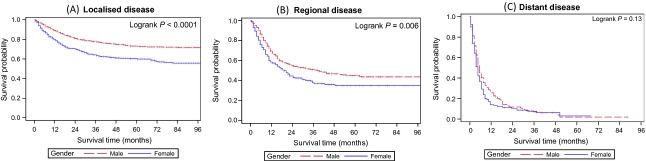
Kaplan–Meier disease-specific survival curves for localized (A), regional (B), and distant (C) disease by Sex for patients aged 70 years or older.

An analysis of 5-year DSS between males and females categorized by age revealed (Fig.3) that there was no difference in DSS between males and females aged less than 70 years (Fig.4). The difference in DSS between females and males was seen entirely in those aged 70 or older (*P* < 0.05) and this older age group accounted for 75% of the female BC diagnoses and 65% of male BC diagnoses.

**Figure 5:**
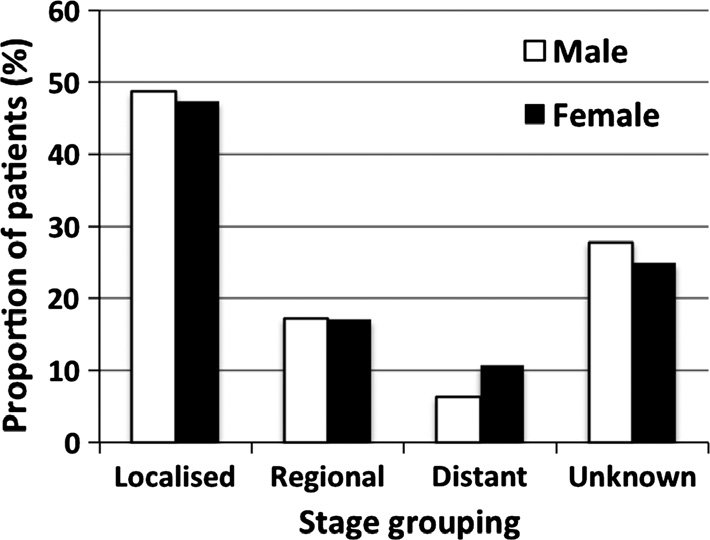
Stage of presentation for patients aged 70 years or older.

We limited further investigation to the cohort of patients aged 70 years or older. There were no differences between the genders in the proportion of patients aged ≥70 years presenting with localized and regional disease (Fig.5). To further determine factors that affect DSS and OS in this older age group, we performed multivariable analysis of potential clinicopathologic and sociodemographic variables (Table2). After controlling for stage, age, comorbidity, and treatment differences, female sex was associated with a 27% inferior DSS from BC in patients aged ≥70 years. Other independent factors associated with poorer DSS included age (HR: 1.07 95% CI 1.06–1.08); regional spread stage at presentation (HR: 2.36 95% CI 2.04–2.74) and distant spread (HR: 7.65, 95% CI 6.48–9.04) compared to the localized spread. CCI of 1 (HR: 1.27, 95% CI 1.09–1.48) and CCI of ≥2 (HR: 1.89, 95% CI 1.68–2.14) were also independently associated with worse DSS. Having treatment in a private hospital (compared to a public hospital) was also associated with substantially better DSS (HR: 0.67, 95% CI 0.60–0.75).

**Table 2:** Bivariate and multivariate analysis of factors determining 5 year DSS and OS in patients with bladder cancer over the age of 70 years

	DSS		OS	
	Bivariate (95% CI)	Multivariate (95% CI)	Bivariate (95% CI)	Multivariate (95% CI)
Sex (female)	1.51 (1.35–1.68)	1.27 (1.14–1.43)	1.19 (1.09–1.29)	1.03 (0.95–1.12)
Age	1.06 (1.05–1.07)	1.07 (1.06–1.08)	1.07 (1.06–1.08)	1.07 (1.07–1.08)
Charlson comorbidity				
0	1	1	1	1
1	1.44 (1.24–1.68)	1.27 (1.09–1.48)	1.56 (1.40–1.74)	1.4 (1.25–1.56)
≥2	2.15 (1.91–2.42)	1.89 (1.68–2.14)	2.35 (2.15–2.57)	2.07 (1.89–2.27)
Stage				
Localized	1	1	1	1
Regional	2.37 (2.06–2.74)	2.36 (2.04–2.74)	1.66 (1.48–1.85)	1.68 (1.50–1.88)
Distant	8.50 (7.24–9.97)	7.65 (6.48–9.04)	5.31 (4.63–6.10)	4.84 (4.20–5.58)
Unknown	1.35 (1.17–1.56)	1.29 (1.12–1.49)	1.29 (1.17–1.42)	1.22 (1.10–1.34)
Cystectomy				
Yes	1	1	1	1
No	1.06 (0.90–1.24)	0.91 (0.76–1.08)	1.38 (1.21–1.58)	1.11 (0.97–1.29)
Intravesical therapy				
Yes	1	1	1	1
No	2.45 (1.96–3.07)	1.86 (1.48–2.34)	2.02 (1.74–2.35)	1.77 (1.52–2.07)
Intravenous therapy				
Yes	1	1	1	1
No	0.76 (0.62–0.94)	0.83 (0.66–1.03)	0.86 (0.72–1.01)	0.77 (0.65–0.92)
Hospital of primary treatment				
Public	1	1	1	1
Private	0.63 (0.57–0.70)	0.67 (0.60–0.75)	0.68 (0.63–0.73)	0.70 (0.64–0.76)
Socioeconomic status SEIFA				
Least disadvantaged (Quintile 1)	1	1	1	1
Quintile 2	0.85 (0.71–1.02)	0.88 (0.73–1.07)	0.94 (0.82–1.08)	0.97 (0.84–1.11)
Quintile 3	1.07 (0.91–1.26)	1.17 (0.98–1.39)	1.09 (0.97–1.23)	1.17 (1.03–1.33)
Quintile 4	1.01 (0.86–1.19)	1.15 (0.97–1.37)	1.03 (0.91–1.16)	1.12 (0.98–1.27)
Most disadvantaged (Quintile 5)	0.98 (0.82–1.16)	1.03 (0.86–1.25)	1.11 (0.97–1.26)	1.16 (1.01–1.34)
Remoteness of residence (ARIA)				
Major cities	1	1	1	1
Inner regional	0.99 (0.87–1.12)	0.98 (0.85–1.12)	0.99 (0.90–1.09)	0.95 (0.86–1.06)
Outer regional/remote	1.07 (0.86–1.32)	1.03 (0.82–1.29)	1.05 (0.90–1.24)	0.97 (0.82–1.15)

DSS, disease specific survival; OS, overall survival; ARIA, Accessibility/Remoteness Index of Australia.

As it appeared from univariate analysis that localized and regional disease was where the largest gender differences were encountered (Fig.2), a separate multivariable analysis was performed for each stage to determine at which stage gender differences existed. In localized disease, females suffered a 77% worse DSS (HR: 1.77, 95% CI: 1.40–2.25), regional (HR: 1.38, 95% CI: 0.96–1.98) and distant (HR: 0.88, 95% CI: 0.40–1.99) disease did not demonstrate gender differences in DSS.

There was no significant difference in OS between males and females aged ≥70 years and diagnosed with BC. Analysis of independent factors associated with OS revealed that age, stage at presentation, comorbidity, and treatment in a private hospital remained significantly associated with OS.

## Discussion

In this NSW population-wide study, we identified a substantial difference in death rates between females and males (40% vs. 30%), 5 year BC DSS (56.5% vs. 66.0%) and OS rates (47.8% vs. 54.5%). We further observed that this difference was limited to those over 70 years and could not be accounted for by differences in stage at presentation, comorbidities, treatment, SES or remoteness of residence.

Our initial hypothesis was that this could be due to a delay in diagnosis in females resulting in higher stage disease at presentation; as hematuria is the most common initial symptom of BC and in females this is commonly attributed to urinary tract infections. This is supported by a Spanish study which reported females were more likely to have larger or multifocal tumors at diagnosis than males 6. Data on delay in diagnosis was not available for our study, but we did not identify any difference in stage at presentation between the genders in the whole cohort or in those ≥70 years (Figs.1 and 5) suggesting there was no major delay in diagnosis that would have contributed to worse prognosis, although we acknowledge small differences would be difficult to identify from this database.

The issue of gender and survival from BC has been a controversial issue. A number of studies have reported no difference in survival between men and women with BC. Mitra et al. have argued that all the previous analyses are unmatched with disproportionally balanced male patient cohorts 11. In matched analyses of men and women undergoing cystectomy, this group has reported equivalent outcomes between genders. In this study however, the median age of females undergoing cystectomy was 65 years, hence a major difference between genders would not be expected (based on our findings that gender inequity occurs after age 70 years). Another study of patients having cystectomy for pT4 disease did not find any difference in survival between genders 18 and an Austrian group has also reported that proportionally more women present with lower stage disease than men, but if muscle invasion was identified in an individual with BC, women demonstrated worse survival. Overall there was no difference in survival between genders in this study 19.

Our large whole population study has added substantially to the literature and confirms the findings of other studies 4–10 of an inequity in BC survival between genders. The major advantage of whole population studies are that referral and treatment biases are avoided when studying outcomes from BC. A European review of survival from BC in nine countries revealed the 5-year relative survival was 72% in males and 67% in females 20. Supporting this, a large multi-institutional analysis of 8000 patients following radical cystectomy reported that female patients experienced a higher stage and were an independent risk factor for cancer-specific mortality 21. In addition a retrospective study from Netherlands has suggested that females were more likely to be diagnosed with invasive forms of BC compared to males (37% vs. 29%) but this difference reduced with advancing pathological stage and metastatic disease incidence was similar in both groups (2.8% vs. 2.7%) 4,5.

Although others have reported that age and gender separately are independent risk factors for mortality from BC 10, no study has reported a specific age at which the gender difference occurs. Age has been shown to be a strong and independent risk factor for development of BC 22. The mortality to incidence ratios for men and women aged 65–69 years is 14% and 18%, respectively but 30% and 37% for those aged 80–84 years 22. Treatments outcomes for the elderly are poorer than for younger patients and may be due to the fact that intra-vesical therapies are less used because of complications 23 or efficacy 24 in this age group. These poorer outcomes may also be due to older patients not being offered radical treatments such as cystectomy because of age or comorbidities 25. In our study, the proportion of women aged >70 years was substantially higher than men (75% vs. 65%) and the median age of women diagnosed with BC was 77 compared to 74 for men. The older population is a potential explanation for worse survival in women, however a difference between genders was observed in both age groups of 70–79 and 80+ and in addition, after adjusting for age in a multivariable analysis of those over 70 years, gender was still a significant predictor of BC DSS. Increased comorbidities (competing risks) are also another potential explanation for poor survival, but this was adjusted for in our study and differences still existed. A statistical difference in OS was not observed between genders in this study but this observation could be simply explained by the fact that women in Australia have a life expectancy of 4.4 years greater than men and the increased BC mortality in women has resulted in no significant difference in OS between the genders.

Other potential explanations for gender disparity are that women aged over 70 years may have presented with higher stage disease, yet we found that there are no gender differences in stage at presentation in this older age group (Fig.4). In addition, we did not observe a difference in use of intravesical treatments, cystectomy or chemotherapy rates in this older age group so differences in treatment between genders are also an unlikely cause.

One potential explanation for the differential behavior between genders over the age of 70 years can be due to a biologically increased aggressiveness related to sex steroids and their receptors. An epidemiological study shown that postmenopausal women have a higher risk of developing BC than premenopausal women 26. Androgen receptor has been identified in normal bladders 27,28 and laboratory studies have shown that the androgen receptor pathway may be important in the development and progression of BC 29. Estrogen receptor has also been identified in the urothelium and although its function is not yet known, it may have a protective effect which is lost in the postmenopausal female 30.

This study has a number of strengths; firstly it is a population-based study. The publication of large multicenter studies from centres of excellence can be biased in terms of referral patterns, disease spectrum treated, diagnostic, and management patterns and are unlikely to accurately reflect the patterns of care and outcomes in the general community. Population-based outcome studies are therefore important to determine current outcomes, which then provides the ability to implement changes if patterns of care do not meet well-established standards. Secondly, this study includes all patients diagnosed with invasive BC, not just cystectomy series which may be biased with respect to age, comorbidities, and other unmeasured variables. Thirdly, our data captures all histologically diagnosed BC cases in the state, as there is a legal requirement that all new diagnoses of invasive BC in NSW are notified to the NSW cancer registry, resulting in very high rates of ascertainment (100%). In addition, data linkage with all hospital admissions to any private or public hospital was gained (with 98% matching) and linkage to the registry of births and deaths ensures a high quality data set with negligible occurrences of missed data.

The limitations of this current study include its retrospective nature, the lack of centralized pathologic review, potential biases from 26% of patients with unknown stage and limited pathological and staging information. The NSW CCR only collects data on invasive BC (pT1 and higher) so the analysis and conclusions are limited to this sub-set of BCs. The accuracy of treatment details relies on accurate coding in the treating hospitals.

Future research should include the investigation of the role of estrogen and estrogen receptors in BC and further epidemiological analysis with centralized pathologic review and more detailed staging and treatment analysis.

## Conclusions

This population-based observational study has demonstrated that there is a clear inequity in survival for women diagnosed with invasive BC, which is localized or spread regionally compared to men. The difference is limited to patients over the age of 70 years and no differences are seen between the genders when younger than 70 years. In patients aged 70 years and over, being female remained an independent risk factor for higher DSS in addition to age and stage and comorbidity. Although robust explanations for this worse survival remain elusive, these observations raise the hypothesis that BC in older females may be biologically different to males.

## Conflict of Interest

None declared.
